# Prompt treatment-seeking behaviour varies within communities among guardians of children with malaria-related fever in Malawi

**DOI:** 10.1186/s12936-023-04680-6

**Published:** 2023-08-26

**Authors:** Christopher C. Stanley, James Chirombo, Harrison Msuku, Vincent S. Phiri, Noel Patson, Lawrence N. Kazembe, Jobiba Chinkhumba, Atupele Kapito-Tembo, Don P. Mathanga

**Affiliations:** 1 MAC-Communicable Diseases Action Centre, Kamuzu University of Health Sciences, Chichiri, Private Bag 360, Blantyre, Malawi; 2 Malawi-Liverpool-Wellcome Clinical Research Programme, Blantyre, Malawi; 3 School of Global and Public Health, Kamuzu University of Health Sciences, Blantyre, Malawi; 4 Department of Computing, Mathematical and Statistical Sciences, University of Namibia, Windhoek, Namibia

**Keywords:** Malawi, Malaria, Fever, Prompt treatment-seeking

## Abstract

**Background:**

In Malawi, malaria is responsible for 40% of hospital deaths. Prompt diagnosis and effective treatment within 24 h of fever onset is critical to prevent progression from uncomplicated to severe disease and to reduce transmission.

**Methods:**

As part of the large evaluation of the malaria vaccine implementation programme (MVIP), this study analysed survey data to investigate whether prompt treatment-seeking behaviour is clustered at community-level according to socio-economic demographics.

**Results:**

From 4563 households included in the survey, 4856 children aged 5–48 months were enrolled. Out of 4732 children with documented gender, 52.2% were female and 47.8% male. Among the 4856 children, 33.8% reported fever in the two weeks prior to the survey. Fever prevalence was high in communities with low socio-economic status (SES) (38.3% [95% CI: 33.7–43.5%]) and low in areas with high SES (29.8% [95% CI: 25.6–34.2%]). Among children with fever, 648 (39.5%) sought treatment promptly i.e., within 24 h from onset of fever symptoms. Children were more likely to be taken for prompt treatment among guardians with secondary education compared to those without formal education (aOR:1.37, 95% CI: 1.11–3.03); in communities with high compared to low SES [aOR: 2.78, 95% CI: 1.27–6.07]. Children were less likely to be taken for prompt treatment if were in communities far beyond 5 km to health facility than within 5 km [aOR: 0.44, 95% CI: 0.21–0.92].

**Conclusion:**

The high heterogeneity in prevalence of fever and levels of prompt treatment-seeking behaviour underscore the need to promote community-level malaria control interventions (such as use of long-lasting insecticide-treated nets (LLINs), indoor residual spraying (IRS), intermittent preventive therapy (IPT), presumptive treatment and education). Programmes aimed at improving treatment-seeking behaviour should consider targeting communities with low SES and those far from health facility.

## Background

Malaria is a major public health problem worldwide with the highest proportion of burden in sub-Saharan Africa (SSA) [1]. In Malawi, malaria is endemic with nearly 4 million people diagnosed with the infection every year, and is one of the leading causes of morbidity and mortality in children [2]. Malawi is among the top 15 countries with a high malaria burden and accounts for 2% of malaria cases worldwide [1]. The World Health Organization (WHO) indicates that malaria diagnosis is suspected primarily on the basis of fever or history of fever in malaria-endemic countries, including Malawi [1, 3]. In such settings, prompt diagnosis and effective treatment of malaria is one of key interventions recommended by the WHO [4]. Fever is a common clinical sign of *Plasmodium falciparum* infection [3, 5–7]. The WHO recommends early malaria diagnosis and treatment i.e., within 24 h of the onset of symptoms [1] to prevent progression from uncomplicated to severe malaria [8, 9] and reduce transmission [10].

In the malaria-endemic SSA region, treatment seeking behaviours among guardians for children under-five years of age with malaria related fever remain suboptimal [11–15]. For example, treatment was sought promptly for 42% of the fever episodes in a longitudinal study in Zambia [15], about 50% in a cross-sectional study in Equatorial Guinea [12], 60% in demographic and household survey in Mozambique [14]. Documented factors associated with treatment-seeking behaviour include: guardian education level, distance to nearest health facility, preferred source of treatment and overall knowledge about malaria [16].

In Malawi, previous studies have shown that levels of prompt treatment-seeking among caregivers of under-five children are also low, hovering around 46% [16, 17]. Majority of studies examining treatment-seeking behaviour have however been limited in two areas: (a) assessing the general treatment-seeking behaviour, without considering the promptness at which the treatment was sought, (b) assessing the behaviour at national level with limited data on community-level factors to inform district level decision making. The WHO has recently highlighted the key strategy to reignite progress to control malaria, the “High burden to high impact” (HBHI) approach [18] which aims to move away from a “one-size-fits all” interventions, promoting tailored responses based on local data and intelligence [1]. In order to fill this knowledge gap, as part of a large evaluation of Malaria Vaccine Implementation Programme (MVIP) in Malawi, this study investigated whether prompt treatment-seeking behaviour is clustered spatially at community-level according to demographic and socio-economic characteristics.

## Methods

### Study site

**Fig. 1:**
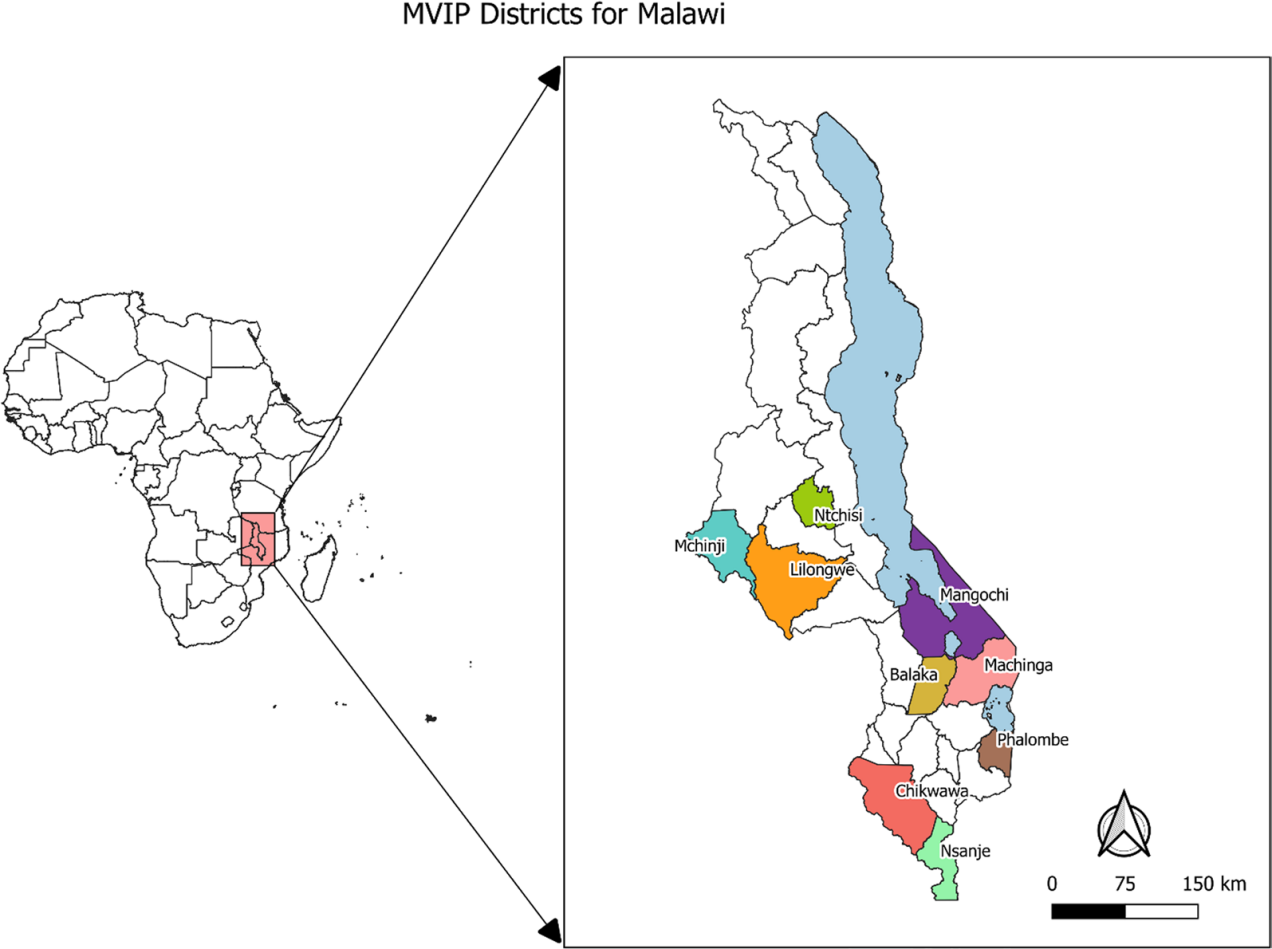
Map of Malawi showing districts implementing the malaria vaccine implementation program (MVIP)

This study analysed data from a community household survey under the malaria vaccine implementation program (MVIP) in Malawi. The MVIP survey was conducted in nine (9) districts providing the RTS,S/A01 malaria vaccine on a pilot basis. The nine districts are spread across the central (Ntchisi, Lilongwe rural, Mchinji) and southern (Balaka, Mangochi, Machinga, Phalombe, Chikwawa, Nsanje) regions of Malawi (Fig. 1). The districts were selected based on the intensity of malaria transmission and the burden of malaria disease. In all these districts, malaria transmission is intense and year-round, peaking during the rainy season (November–May). This survey was conducted in May–June 2019. *Plasmodium falciparum* is the dominant parasite species although *Plasmodium malariae* and *Plasmodium ovale* have also been recorded [19]. *Anopheles funestus* is the main vector for malaria transmission in the area and high levels of pyrethroid resistance have been reported across these districts [19, 20]. During the time of this data collection, the Malawi government was implementing three key interventions: prompt access to artemisinin-based combination therapy (ACT)-defined as treatment with any type of artemisinin-based combination within 24–48 h of fever onset [21]; intermittent preventive treatment during pregnancy (IPTp) and use of long-lasting insecticidal net (LLIN). Free LLINs are distributed to pregnant women at their first antenatal care clinic visit, children born in health facilities and infants attending their first under-five clinic visit. This continuous distribution of LLINs is interspaced with mass distribution campaigns, and the last one prior to this survey was undertaken in October to December, 2018. Furthermore, under community case management, government-employed health surveillance assistants (HSAs) provide treatment for different diseases including malaria, pneumonia, diarrhoea and eye infections in village clinics. Treatment of malaria is based on a malaria blood test (mRDT and/or blood smear) and both the tests and treatment artemisinin-based combinations are provided for free in public health facilities and village clinics.

### Study design

The household survey was conducted in nine (9) districts across Malawi which were partitioned into 46 clusters. A two-stage cluster design sampling was used to select 100 households in each cluster. Firstly, forty-six (46) clusters, each with a population of at least 130,000 people and an estimated population of 4000 children aged 5–48 months, were created in the nine districts. In each cluster, four (4) enumeration areas (EAs) defined as a census track with an average of population of 1200 people (235–1000 households) were selected based on probability proportional to estimated population size (PPS). A total of 184 EAs were selected in which all households with at least one eligible child aged 5–48 months served as the sampling frame. For the second stage, a fixed number of 35 households with children aged 5–48 months were then randomly selected in each EA and trained data collectors collected data from the first 25 households. In the selected households, children aged 5–48 month were enrolled if guardians provided informed consent. The current analyses included data for 4856 children.

### Outcome

The primary outcome of this study was prompt treatment-seeking behaviour defined as when a guardian sought treatment for a child within 24 h from onset of fever symptoms, as per the WHO malaria treatment guidelines [21]. In this study, fever is defined based on guardian reports to within 2 weeks preceding the survey.

### Covariates

The primary covariate was Euclidean distance in kilometres (kms) calculated from the household to the nearest health facility (HF). Households within a radius of 5 km of the nearest HF were considered to be easily accessible as per WHO’s recommendation [22, 23]. Other covariates considered included age and gender of the child, guardians’ age, gender, education level, religion and marital status; and socio-economic status (SES) of the households. The SES indicator was created by combining ownership of household belongings and applying principal component analysis (PCA) technique [24]. The PCA assigned individual households on a continuous scale of relative wealth, and then categorized on the following levels: low (i.e., lower/lowest), middle and high (i.e., higher/highest). Community level variables were constructed by aggregating individual-level factors.

### Statistical analysis

Baseline characteristics were summarized using weighted numbers (and proportions) to account for the clustering nature of the data. The descriptive statistics were disaggregated by the primary exposure (distance from the household to the nearest HF) and compared across groups using chi-squared test. Multilevel logistic regression models were also fitted in order to assess the association between prompt treatment-seeking behaviour and different covariates including distance to health facility, individual factors (age and gender of child, guardian’s level of education, marital status and religion) and community-level factor (socio-economic status). Covariates were included in the final adjusted model if they were significant in unadjusted model at a conservative alpha-level of 0.1. A generalized binomial geostatistical model with intercept only was also fitted on prompt treatment-seeking behaviour as the outcome to provide smoothed maps and hence estimate unadjusted proportions that sought treatment in areas that were not sampled. To achieve this, modelled estimates were used to predict prevalence at unsampled locations. Regular 1 km × 1 km grids were created covering the entire areas of the study districts to provide higher resolution estimates of prompt treatment-seeking behaviour in unsampled locations. During prediction, the model parameters were fixed at their converged values. The predictive distribution of prevalence were summarized through mean in each grid [25]. Descriptive statistics and logistic regression modelling were done using Stata SE version 15.1 (Stata Corp., College Station, TX) [26]. The estimation of the parameters in the geostatistical model was done via Monte Carlo Maximum Likelihood (MCML) implemented in R [27] version 4.0.3 using the package *PrevMap* [25]*.* Statistical significance was considered at a two-sided alpha level of 0.05.

## Results

### Demographic and socio-economic characteristics

There were 4856 children aged 5–48 months enrolled from 4563 households. Out of the 4856 children, 631 (13.0%) were 5–11 months, 1464 (30.2%) were 12–23 months and 2764 (56.9%) were 24–48 months. Out of 4732 children with documented gender, 2472 (52.2%) were female and 2260 (47.8) were male. Close to half, 2272 (49.6%) of the guardians were aged 20–29 years, followed by those aged 30–39 years 1,157 (25.3%), 40–49 years 306 (6.7%) and older than 50 years 78 (1.7%). Out of all 4, 576, 673 (14.7%) had no formal education, 3113 (68.0%) had attained primary education, 742 (16.2) secondary and 48 (1.1%) tertiary education. Out of 4563 households included in the survey, 1372 (30.1%) were from Lilongwe, 800 (17.5%) Mangochi, 600 (13.2%) Machinga, 493 (10.8%) Mchinji, 399 (8.7%) Balaka, 200 (4.4%) for Chikwawa and Nsanje each, and 199 (4.4) Ntchisi. Of these households, 4,431 (97.1%) were in rural and 132 (2.9%) were in in urban. Out of the 46 clusters, 27 (58.7%) were predominantly low, 5 (10.9%) moderate and 14 (30.4%) high SES (Table 1).

**Table 1:** Demographic and socio-economic characteristics of children and guardians

Characteristics	All	Within 5 km	Beyond 5 km
*Individual-level factors*			
Age of child in months, n (%)	*(n = 4856)*	*(n = 2738)*	*(n = 2118)*
5–11	631 (13.0)	350 (12.8)	281 (13.3)
12–23	1464 (30.2)	838 (30.6)	626 (29.6)
24–26	396 (8.2)	212 (7.7)	184 (8.7)
27–38	1386 (28.5)	786 (28.7)	600 (28.3)
39–48	976 (20.1)	551 (20.1)	425 (20.1)
Gender of child, n (%)	*(n = 4732)*	*(n = 2660)*	*(n = 2072)*
Female	2472 (52.2)	1422 (53.5)	1050 (50.7)
Male	2260 (47.8)	1238 (46.5)	1022 (49.3)
*Guardian characteristics*			
Gender of guardian, n (%)	*(n = 4576)*	*(n = 2575)*	*(n = 2001)*
Female	4363 (95.3)	2440 (94.8)	1923 (96.1)
Male	213 (4.7)	135 (5.2)	78 (3.9)
Age of guardian in years, n (%)	*(n = 4189)*	*(n = 2354)*	*(n = 1835)*
15–19	376 (8.2)	202 (7.8)	174 (8.7)
20–29	2272 (49.6)	1243 (48.3)	1029 (51.4)
30–39	1157 (25.3)	663 (25.8)	494 (24.7)
40–49	306 (6.7)	187 (7.3)	119 (5.9)
50 +	78 (1.7)	59 (2.3)	19 (1.0)
Education level of guardian, n (%)	*(n = 4576)*	*(n = 2575)*	*(n = 2001)*
No education	673 (14.7)	380 (14.8)	293 (14.6)
Primary	3113 (68.0)	1690 (65.6)	1423 (71.0)
Secondary	742 (16.2)	464 (18.0)	278 (13.9)
Tertiary	48 (1.1)	41 (1.6)	7 (0.4)
Marital status for guardian, n (%)	*(n = 4576)*	*(n = 2575)*	*(n = 2001)*
Married	3786 (82.7)	2055 (79.8)	1731 (86.4)
Widowed	102 (2.2)	73 (2.8)	29 (1.5)
Divorced/separated	541 (11.8)	331 (12.9)	210 (10.5)
Never married	122 (1.7)	97 (3.8)	25 (1.3)
Other	25 (0.6)	19 (0.7)	6 (0.3)
Religion, n (%)	*(n = 4576)*	*(n = 2575)*	*(n = 2001)*
CCAP	531 (11.6)	262 (10.2)	269 (13.4)
Catholic	594 (594)	364 (14.1)	230 (11.5)
Anglican	109 (2.4)	81 (3.2)	28 (1.4)
SDA	184 (4.0)	101 (3.9)	83 (4.1)
Muslim	1310 (28.6)	877 (34.1)	433 (21.6)
No religion	60 (1.3)	31 (1.2)	29 (1.5)
Apostolic	83 (1.8)	55 (2.1)	28 (1.4)
Jehovah’s witness	75 (1.6)	37 (1.4)	38 (1.9)
Other	1630 (35.6)	767 (29.8)	863 (43.1)
*Household characteristics*			
District of residence, n (%)	*(n* = *4563)*	*(n* = *2569)*	*(n* = *1994)*
Ntchisi	199 (4.4)	174 (6.8)	25 (1.3)
Lilongwe rural	1372 (30.1)	220 (8.6)	1152 (57.8)
Mchinji	493 (10.8)	312 (12.1)	181 (9.1)
Balaka	399 (8.7)	338 (13.2)	61 (3.1)
Mangochi	800 (17.5)	598 (23.3)	202 (10.1)
Machinga	600 (13.2)	335 (13.0)	265 (13.3)
Phalombe	300 (6.6)	249 (9.7)	51 (2.6)
Chikwawa	200 (4.4)	143 (5.6)	57 (2.9)
Nsanje	200 (4.4)	200 (7.8)	0 (0)
Size of household, n (%)	*(n* = *4563)*	*(n* = *2569)*	*(n* = *1994)*
≤ 4 members	2316 (50.8)	1237 (48.2)	1079 (54.1)
> 4 members	2247 (49.2)	1332 (51.8)	915 (45.9)
*Community (cluster) level factors*			
Socio-economic, n (%)	*(n* = *46)*	*(n* = *31)*	*(n* = *15)*
Lower/lowest	27 (58.7)	16 (51.6)	11 (73.3)
Middle	5 (10.9)	3 (9.7)	2 (13.3)
Higher/highest	14 (30.4)	12 (38.7)	2 (13.3)

^+^Based on guardian education level

### Prevalence of febrile malaria and prompt treatment-seeking behaviour

Out of 4,856 children 1, 639 (33.8% [95% CI: 31.4–36.2%]) had fever in the two weeks prior to the survey. Overall, the prevalence of fever was highest in Balaka district (44.9% [95% CI: 39.0–50.9%]) followed by Mchinji (38.4% [95% CI: 29.3–48.4%]), and was lowest in Nsanje (21.0% [95% CI: 17.3–25.2%]) followed by Phalombe (23.1% [95% CI: 13.7–36.3%]). Fever was reported more among children aged 27–38 months (35.9% [95% CI: 32.3–39.7%]), and lowest among 39–48 months old (31.2% [95% CI: 27.2–36.8%]) (Table 2). Based on guardian’s age, fever was highest among children who were under guardians aged over 50 years (48.9% [95% CI: 24.0–74.3%]) and lowest among children whose guardians were aged 30–39 years (29.8% [95% CI: 25.8–34.2%]). The prevalence of fever was higher among households with low socio-economic status (38.3% [95% CI: 33.7–43.5%]) compared to households with high socio-economic status (29.8% [95% CI: 25.6–34.2%]).

**Table 2:** Health-seeking behaviour among children aged 5–48 months with history of fever

Characteristics	Sample	Percent with fever * (95% CI)	Percent who sought treatment within 1 day among those with fever (95% CI)
Gender of child			
Female	2472	32.3 (29.2, 35.5)	38.5 (34.7, 42.5)
Male	2260	36.2 (33.5, 39.0)	39.3 (35.3, 43.5)
Age of child in months			
5–11	631	32.7 (28.2, 37.5)	41.7 (34.0, 49.9)
12–23	1464	34.3 (30.7, 38.0)	36.8 (31.7, 42.2)
24–26	396	31.8 (24.4, 38.9)	37.7 (26.7, 50.2)
27–38	1386	35.9 (32.3, 39.7)	38.0 (32.8, 43.5)
39–48	976	31.2 (27.2, 36.8)	42.8 (34.9, 51.1)
Gender of guardian			
Female	4363	34.3 (31.9, 36.7)	39.4 (35.6, 43.4)
Male	213	24.8 (11.9, 44.6)	35.1 (12.8, 66.5)
Guardian’s age in years			
15–19	376	39.0 (31.8, 46.8)	35.7 (26.8, 43.7)
20–29	2272	34.3 (31.5, 37.1)	41.6 (35.3, 48.2)
30–39	1157	29.8 (25.8, 34.2)	40.6 (33.3, 48.3)
40–49	306	43.1 (32.3, 54.5)	59.9 (45.1, 74.7)
50 +	78	48.9 (24.0, 74.3)	12.5 (2.5, 30.7)
Guardian’s level of education			
No education	673	33.5 (29.5, 37.8)	32.9 (24.8, 38.9)
Primary	3113	35.5 (32.9, 38.3)	39.2 (34.5, 44.1)
Secondary	742	29.7 (25.8, 34.0)	47.7 (40.2, 55.8)
Tertiary	48	19.9 (10.1, 35.6)	33.7 (8.4, 73.8)
Marital status for guardian			
Married	3786	33.7 (31.3, 36.2)	38.9 (35.7, 42.2)
Widowed	102	24.9 (16.2, 36.3)	35.0 (20.0, 53.7)
Divorced/separated	541	35.2 (30.0, 40.7)	41.2 (32.9, 50.2)
Never married	122	35.6 (26.3, 46.1)	35.3 (21.0, 52.9)
Other	25	39.3 (21.4, 60.7)	39.6 (13.0, 74.3)
Religion			
CCAP	531	30.2 (25.7, 35.1)	47.7 (37.4, 60.3)
Catholic	594	32.5 (27.7, 37.6)	42.5 (32.5, 51.9)
Anglican	109	31.5 (24.3, 39.6)	52.5 (28.4, 80.2)
SDA	184	25.3 (16.1, 37.4)	42.2 (27.5, 71.6)
Muslim	1310	34.9 (30.6, 39.6)	35.5 (27.7, 37.5)
No religion	60	39.4 (25.8, 54.9)	20.5 (6.1, 50.5)
Apostolic	83	36.3 (24.7, 49.8)	58.4 (34.5, 78.9)
Jehovah’s witness	75	22.9 (13.6, 35.9)	34.2 (15.2, 60.0)
Other	1630	37.1 (33.7, 40.6)	38.1 (32.2, 44.3)
Distance to health facility			
≤ 5 km	2738	32.2 (29.0, 35.5)	41.6 (39.7, 44.5)
> 5 km	2118	35.9 (32.5, 39.4)	35.4 (32.2, 38.5)
Size of household			
≤ 4 members	2316	33.9 (31.1, 37.0)	40.9 (36.7, 45.3)
> 4 members	2247	33.6 (30.6, 36.6)	37.2 (34.6, 41.7)
District of residence			
Ntchisi	199	34.1 (26.7, 42.4)	48.8 (42.2, 55.4)
Lilongwe rural	1372	34.2 (30.0, 38.5)	34.2 (30.0, 38.5)
Mchinji	493	38.4 (29.3, 48.4)	44.5 (29.8, 60.2)
Balaka	399	44.9 (39.0, 50.9)	49.7 (40.3, 59.1)
Mangochi	800	37.0 (33.5, 40.6)	37.0 (33.5, 40.6)
Machinga	600	30.0 (24.4, 36.4)	30.0 (24.4, 36.4)
Phalombe	300	23.1 (13.7, 36.3)	23.1 (13.7, 36.3)
Chikwawa	200	37.4 (26.7, 49.5)	37.4 (26.7, 49.5)
Nsanje	200	21.0 (17.3, 25.2)	41.0 (21.3, 64.0)
Community (cluster) level wealth ranking (n = 46)			
Lower/lowest	27	35.6 (33.1, 38.3)	37.1 (33.4, 43.2)
Middle	5	37.9 (34.8, 41.1)	35.7 (29.8, 42.2)
Highest	5	32.0 (29.2, 34.9)	49.9 (43.9, 55.9)

*Proportion out of the sample expressed as percentage

### Factors associated with prompt treatment-seeking behaviour

Out of the 1,639 children aged 5–48 months who had fever in the two weeks prior to the survey, treatment was sought promptly in 648 (39.5%). Prompt treatment-seeking behaviour varied by age of a guardian being significantly high among those aged 40–49 years (59.9% [95%CI: 45.1–77.7%]) vs 12.5% (95% CI; 2.5–30.7%) among guardians above 50 years and 35.7% (95% CI: 26.8–43.7%) in those aged 15–19 years (Table 2). There was an increasing trend in prompt seeking behaviour with increased level of formal education for a guardian, 32.9% (95% CI: 24.8–38.9%) for those without formal education, 39.2% (95% CI: 34.5%, 44.1%) with primary education, and 47.7% (95% CI: 40.5–55.8%) among guardians with secondary education, but decreased among guardians with tertiary education 33.7% (95% CI: 8.4–73.8%) (Table 2). Among the nine districts covered in this survey, prompt treatment-seeking behaviour was highest in Phalombe (62.2% [95% CI: 50.2–72.9%]) and lowest in Mangochi (28.5% [95% CI: (23.4–34.2%]). Prompt treatment-seeking behaviour was also lower among Muslim guardians (32.3% (95% CI: 27.3%, 37.5%) when compared to other religions such as Anglican (61.0% [95% CI: 37.6%, 80.2%]) or Seventh Day Adventist (SDA) (57.5 [95% CI: 42.0%, 71.6%]).

At community-level, guardians from communities with high proportion of households with high socio-economic status (SES) were more likely to take their children for fever treatment than communities predominantly with households in low SES [aOR: 2.85, 95% CI: 1.30–6.22]. (Table 3). Children from communities with most households beyond 5 km from health facility were less likely to be taken for prompt treatment compared to those where low percentage of households were within 5 km of a health facility [aOR: 0.44, 95% CI: 0.21–0.92].

**Table 3:** Multilevel logistic regression analysis of individual and community-level factors associated with prompt treatment-seeking behaviour

Variable	Unadjusted			Adjusted		
	Odds ratio	(95% CI)	p-value	Odds ratio	(95% CI)	p-value
District of residence						
Ntchisi	1			1		
Lilongwe rural	0.15	(0.03, 0.82)	0.02	0.19	(0.03, 1.08	0.05
Mchinji	0.88	(0.14, 5.34)	0.89	0.97	(0.16, 5.88)	0.97
Balaka	0.67	(0.11, 4.15)	0.67	0.69	(0.11, 4.21)	0.69
Mangochi	0.05	(0.01, 0.34)	< 0.01	0.06	(0.01, 0.37)	< 0.01
Machinga	0.23	(0.04, 1.35)	0.10	0.25	(0.04, 1.49)	0.13
Phalombe	2.17	(0.27, 17.07)	0.46	0.22	(0.29, 16.75)	0.45
Chikwawa	2.63	(0.31, 22.21)	0.37	2.77	(0.34, 22.86)	0.35
Nsanje	0.81	(0.06, 10.17)	0.87	0.08	(0.07, 9.69)	0.86
Religion						
CCAP	1			1		
Catholic	0.87	(0.21, 3.53)	0.85	0.75	(0.18, 3.16)	0.69
Anglican	3.14	(0.25, 39.10)	0.37	2.32	(0.17, 31.41)	0.53
SDA	1.71	(0.23, 12.64)	0.59	1.72	(0.23, 13.47)	0.60
Muslim	0.21	(0.06, 0.74)	0.02	0.17	(0.05, 0.64)	0.01
No religion	0.42	(0.02, 8.73)	0.57	0.37	(0.02, 8.14)	0.53
Apostolic	3.04	(0.20, 45.98)	0.42	2.79	(0.18, 14.34)	0.47
Jehovah’s witness	0.42	(0.02, 8.72)	0.58	0.39	(0.02, 8.66)	0.55
Other	0.51	(0.16, 1.62)	0.25	0.50	(0.15, 1.65)	0.26
Age of guardian (years)						
15–19	1			1		
20–29	2.82	(0.87, 9.11)	0.08	2.84	(0.88, 9.18)	0.10
30–39	2.97	(0.82, 10.73)	0.09	2.99	(0.83, 10.77)	0.13
40–49	9.68	(2.43, 15.05)	0.005	19.08	(2.37, 15.36)	0.005
50 +	0.21	(0.04, 1.29)	0.47	0.22	(0.03, 1.36)	0.48
Education level of guardian						
No education	1			1		
Primary	2.18	(0.82, 5.81)	0.12	2.22	(0.82, 6.00)	0.12
Secondary	12.86	(3.05, 54.18)	< 0.01	12.87	(2.98, 35.62)	< 0.01
Tertiary	3.38	(0.03, 36.1)	0.61	2.61	(0.02, 32.37)	0.69
Community socio-economic status						
Lower/lowest	1			1		
Middle	1.06	(0.55, 1.44)	0.26	1.09	(0.45, 1.79)	0.76
Higher/highest	3.22	(1.33, 7.15)	0.01	2.85	(1.30, 6.22)	0.01
Community distance to health facility						
≤5 km	1			1		
> 5 km	0.53	(0.28, 0.97)	0.04	0.44	(0.21, 0.92)	0.03

*Random intercepts included at the cluster, enumeration area (EA) and household levels

+Guardian education as proxy

### Spatial patterns of prompt treatment-seeking behaviour

**Fig. 2:**
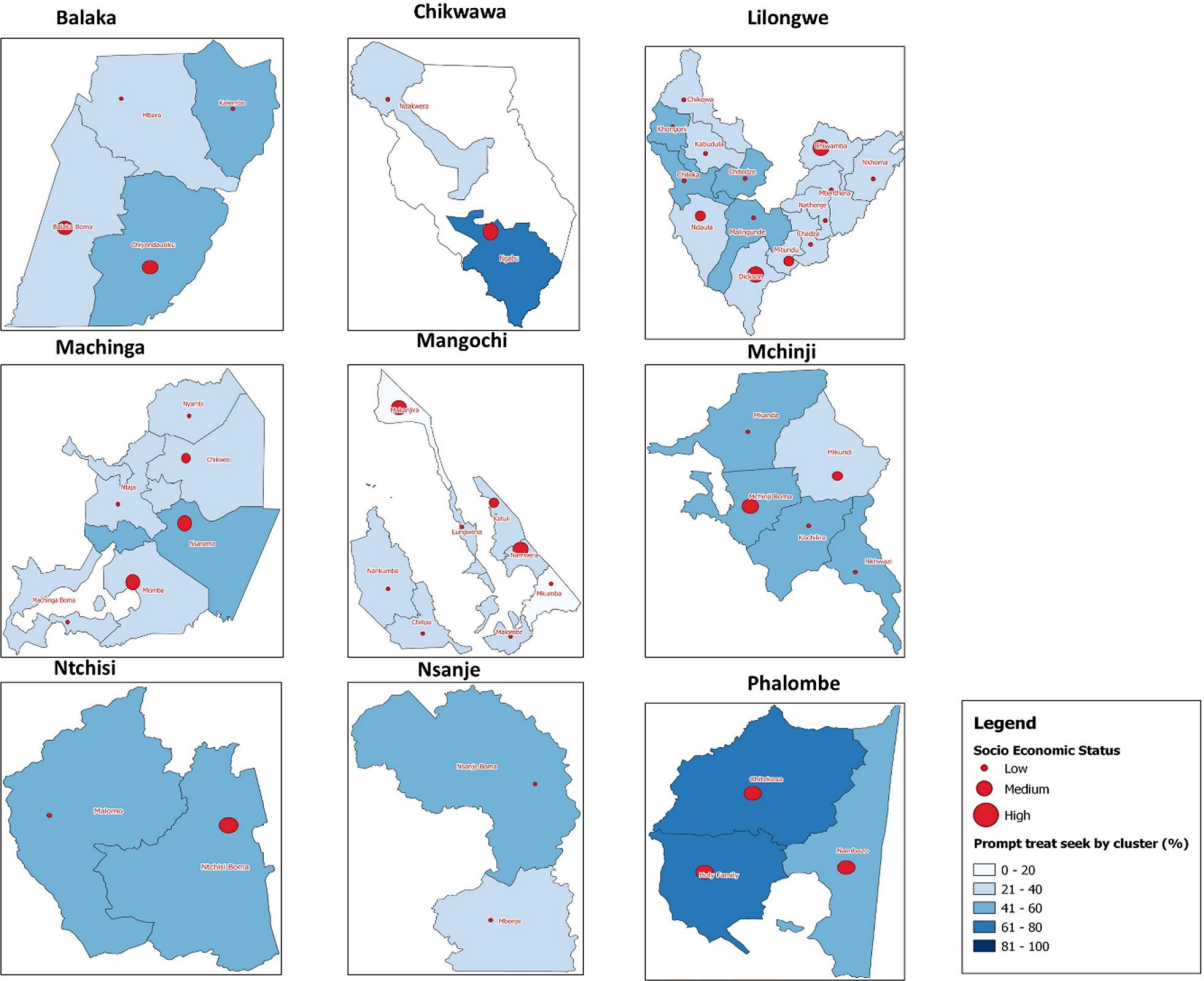
Map showing cluster-level socio-economic status and percentages of prompt treatment seeking behaviours among guardians of children with fever MVIP districts in Malawi

There was variation in proportion of prompt treatment-seeking behaviours for fever across communities (clusters) both within and across the districts (Fig. 2). The proportions of prompt treatment-seeking behaviour varied. Clusters with high SES displayed higher trends in prompt treatment-seeking behaviours compared those with low SES. However, the Freeman’s theta test to check for association between the ordinal SES and nominal prompt treatment seeking behaviour indicated that they were not associated. For all the districts, the theta values were low, ranging from 0.01 to 0.09.

**Fig. 3:**
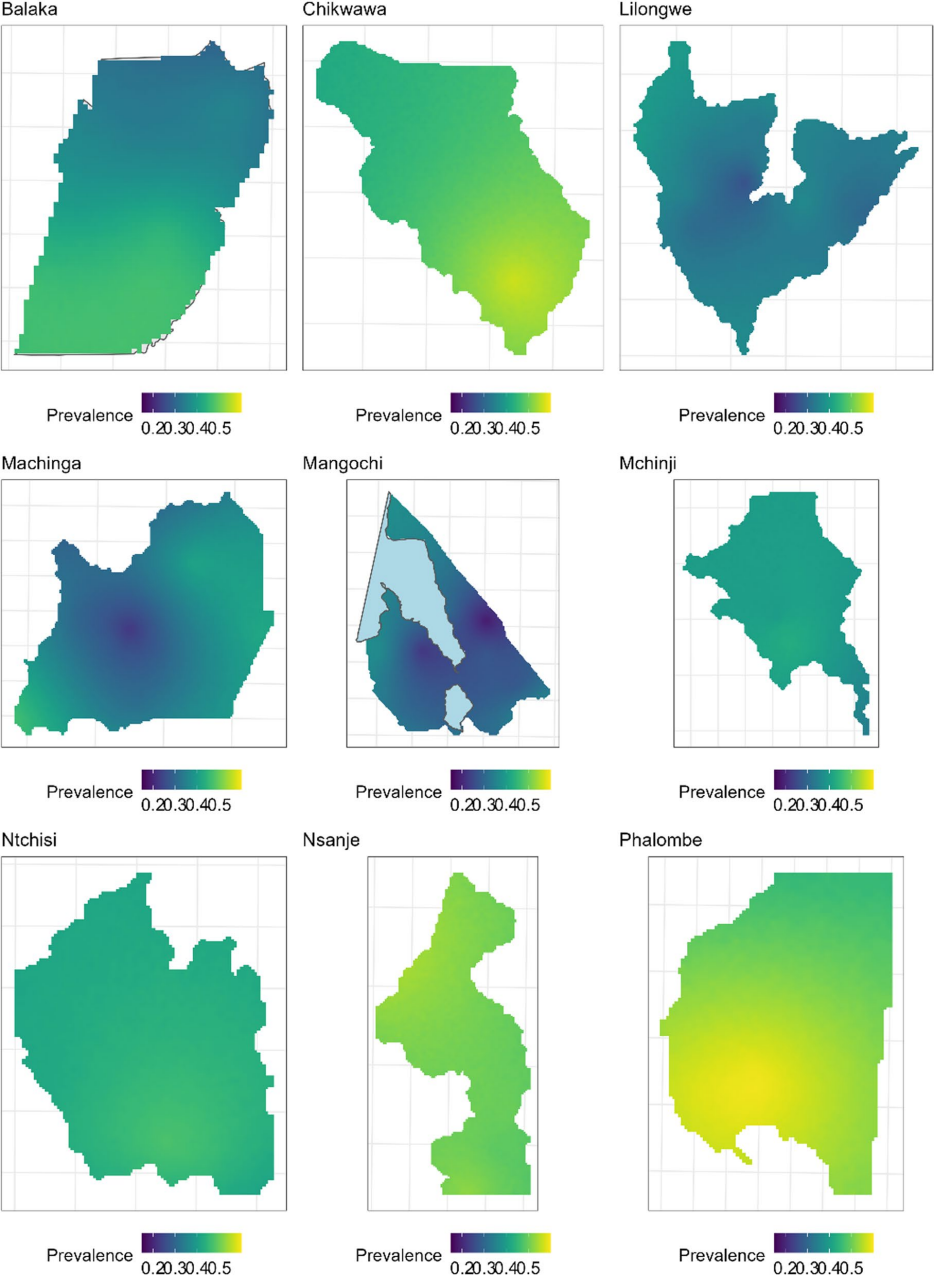
District maps showing smoothed prevalence estimates of prompt treatment-seeking (low in blue areas and high in yellow areas) among children under 5 years with history of fever

Based on the smoothed maps in Fig. 3, there were observed different patterns of prompt treatment-seeking rates across the different districts. This is shown by the hot spots (concentrations of high rates) and cold spots (concentrations of low rates) for prompt treatment seeking behaviour. Overall, higher rates are observed in the districts Balaka, Chikwawa and Nsanje. In Mangochi and Machinga districts, the rates are relatively low but with clear coldspots. In Mangochi, for example, there are two locations of very low rates. Lilongwe, Mchinji, Balaka and Ntchisi districts also exhibit homogeneous relatively low rates across the districts. The treatment-seeking patterns observed in this survey suggest that distance (and how difficult) the individuals travel to an HF determines the behaviour. For example, close examination of physical features in Mangochi show that very cold spots lie between lakes Malawi and Malombe.

## Discussion

This study has demonstrated that prompt treatment-seeking behaviour among guardians of children with malaria related fever remain suboptimal, below 40%. There was heterogeneity in patterns for both the prevalence of fever and prompt treatment-seeking behaviour at cluster-level, i.e., communities even within the same district. Based on maps in Fig. 2, the patterns of prompt treatment-seeking behaviour varied spatially at community level according to SES. Guardians from communities (clusters) with low SES were less likely to take their children for prompt treatment when compared to communities with high SES. Poor treatment-seeking behaviours have also been reported from similar settings to this study [12, 28, 29]. Transport costs have been found to be prohibitive during treatment-seeking among families with low SES [12, 28].

Distance to the health centre influenced malaria treatment-seeking behaviours. Children from communities where most of the households were beyond 5 km from the health facility were less likely to have treatment sought for promptly, compared to their counterparts. In rural areas with long distances to health facility, people resort to the most accessible healthcare providers such as drug sellers, the community health workers and traditional healers [12], resulting in delay in seeking effective treatment from health facility [9].

At individual-level, guardian’s education was positively associated with care-seeking behaviour. Guardians with secondary level of education were more likely to seek care for their children than those with no formal education. This is consistent with previous studies in similar settings which found prompt treatment-seeking behaviour to be lower among guardians without formal education [14, 16, 30, 31]. This lower care-seeking behaviour for fever among guardians without formal education may be attributed to low awareness about prevention, diagnostics, and treatment of malaria. The general knowledge about malaria have previously been associated with better prompt treatment-seeking behaviour [11].

There was higher level of prompt treatment-seeking behaviour among guardians of the active reproductive ages of 30–49 years compared to the non-reproductive age groups. This could be attributed to constant messages that these guardians (who were mostly women) receive when they go for other health services such as antenatal care, postnatal and family planning [32]. In addition to programs that seek to promote treatment-seeking behaviours, key malaria control interventions should leverage on the already existing health services that are provided to guardians. Integration of health services has also been shown to be cost-effective particularly in resource limited settings [33–35].

Guardians that belonged to Muslim religion were less likely to take their children for prompt treatment, consistent with a previous by Nkoka et al*.* [16]. This could be attributed to socio-cultural norms as religious beliefs and cultural practices are strongly linked in Malawi [36]. In order to promote prompt treatment-seeking behaviour in such communities, behavioural change messages must address the prevailing local beliefs about causes of fever and general knowledge about malaria.

Strengths of this study include being a two-stage cluster-randomized survey design which allowed for accessing cluster-specific estimates while also ensuring that the results are nationally representative and therefore generalizable to Malawian guardians. Secondly, the level of data missing in this study was minimal. Nevertheless, this study was limited by potential for recall bias on fever as reported by the guardians who may not be accurate on the timing of fever onset. This bias was however minimized by limiting the history of fever to two weeks prior to the survey. Lastly, even though distance from households to nearest health facility was categorized using Euclidean distance, the HF nearest to each household may not always be the preferred and consistently utilized facility. However, this limitation was minimized by assessing fever and treatment-seeking patterns at community level most of which share potential physical barriers to travel such as mountains and rivers.

Future work should also consider assessing the impact of the malaria vaccination on uptake of other vector control interventions and treatment-seeking behaviour in the communities.

## Conclusions

The high heterogeneity in prevalence of fever and prompt treatment-seeking behaviour underscore the need to promote community-level interventions for malaria control, such as use of LLINs, IRS and presumptive treatment. Programmes aimed at improving treatment-seeking behaviours should consider targeting communities with low SES and longer distances to nearest health facility.

## Data Availability

The dataset analysed during the current study is available from the corresponding author on reasonable request.
